# Supplementary material for: The TSPO-NOX1 axis controls phagocyte-triggered pathological angiogenesis in the eye
Source: Nat Commun. 2020 Jun 1;11:2709. doi: 10.1038/s41467-020-16400-8 (PMC7264151; doi:10.1038/s41467-020-16400-8)
Supplement: Supplementary file 1 — Supplementary Information [file 41467_2020_16400_MOESM1_ESM.pdf]

Supplementary information

The TSPO-NOX1 axis controls phagocyte-triggered pathological angiogenesis in the eye

Wolf. et al.

Supplementary Figures 1-13

Supplementary Figure 1

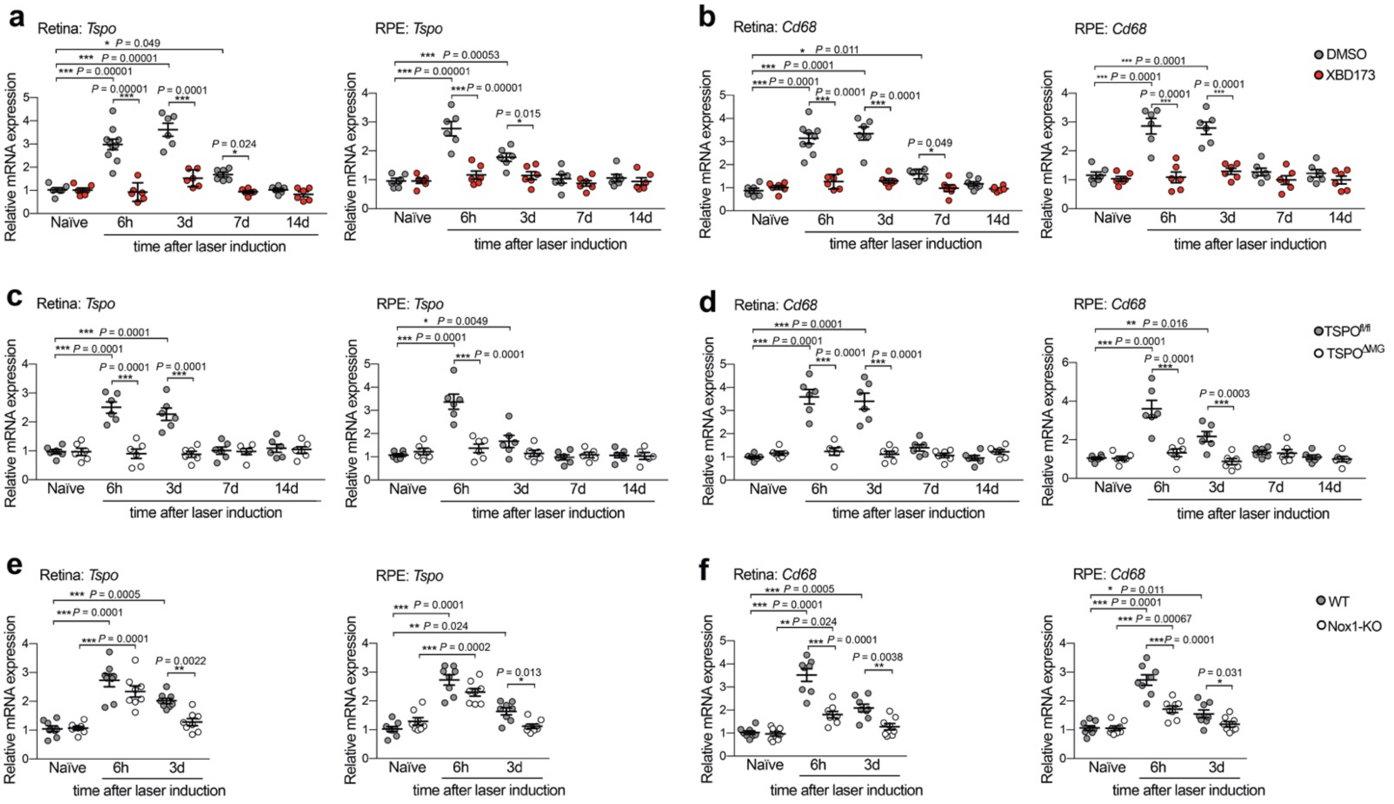

Supplementary Figure 1: Laser-induced gene expression of *Tspo* and *Cd68*. *Tspo* and *Cd68* mRNA levels in retina and RPE/choroid from **a, b** DMSO- or XBD173 treated mice, **c, d** $TSPO^{fl/fl}$ and $TSPO^{\Delta MG}$ mice, and **e, f** WT and Nox1-KO mice at indicated time points after laser-induced CNV. Data are presented as mean ± SEM. n= 6 retinas/RPEs from individual mice, WT/Nox1-KO n= 8 retinas/RPEs from individual mice. Linear mixed model was used for statistical analyses; **P*< 0.05, ***P*< 0.01 and ****P*≤ 0.001. Source data are provided as a Source Data file.

Supplementary Figure 2

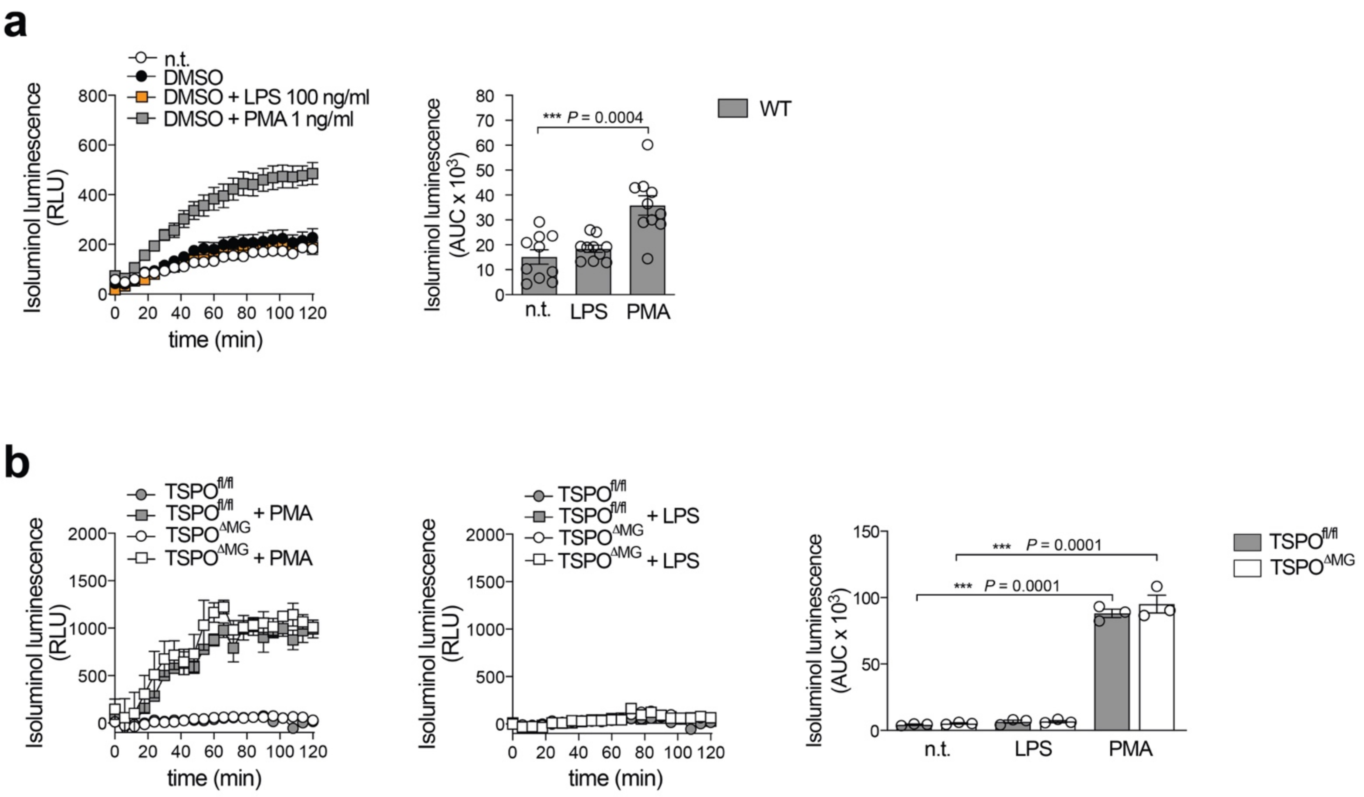

Supplementary Figure 2: Extracellular ROS production is induced in stimulated primary microglia. Primary microglia from WT **(a)**, $TSPO^{fl/fl}$ and $TSPO^{\Delta MG}$ mice **(b)** mice were stimulated with LPS or PMA. Kinetics of ROS production and the area under the curve (AUC) are shown. Data are presented as mean ± SEM. n= 10 (a); 3 (b) independent experiments, two-tailed unpaired Student's *t* test, ****P*≤ 0.001. n.t., non-treated. Source data are provided as a Source Data file.

Supplementary Figure 3

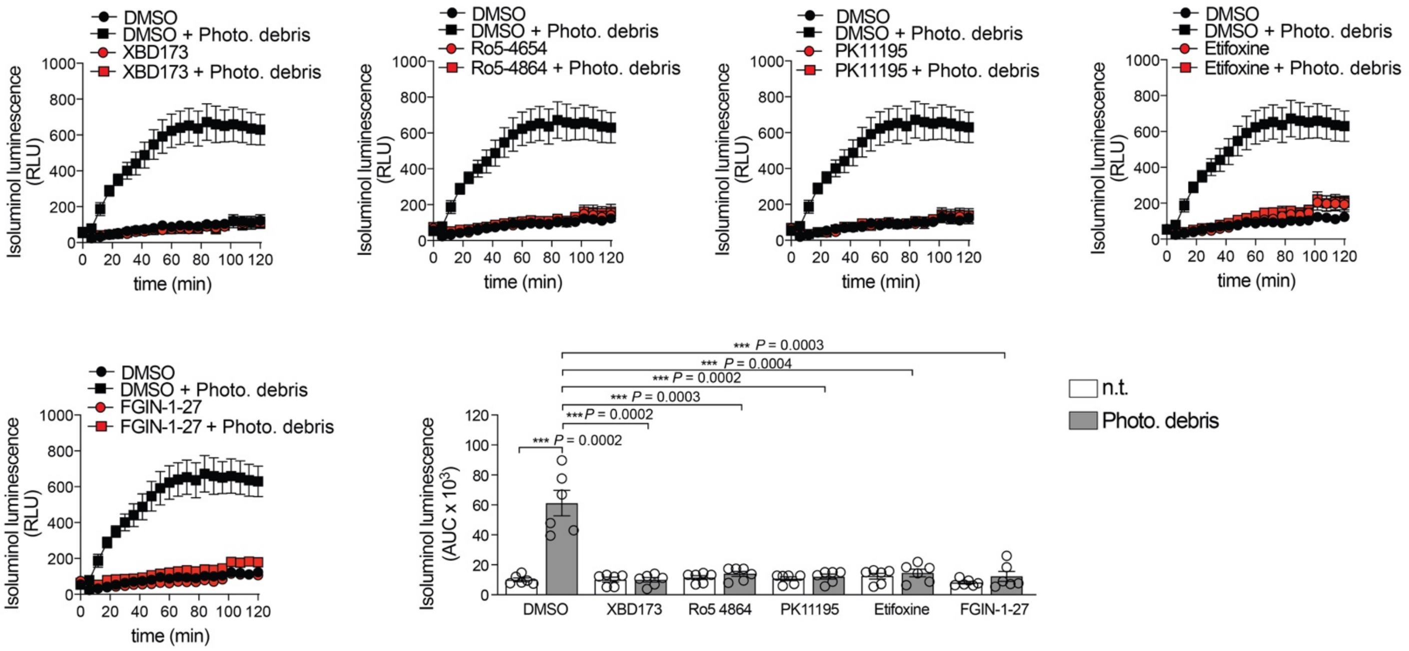

Supplementary Figure 3: TSPO ligands reduce extracellular ROS production in stimulated primary microglia. Primary microglia were stimulated with photoreceptor cell debris and treated with 50 µM of different TSPO ligands. Kinetics of ROS production and the area under the curve (AUC) are shown. Data are presented as mean ± SEM. n= 6 independent experiments, two-tailed unpaired Student's *t* test, ***$P \leq 0.001$. n.t., non-treated. Source data are provided as a Source Data file.

Supplementary Figure 4

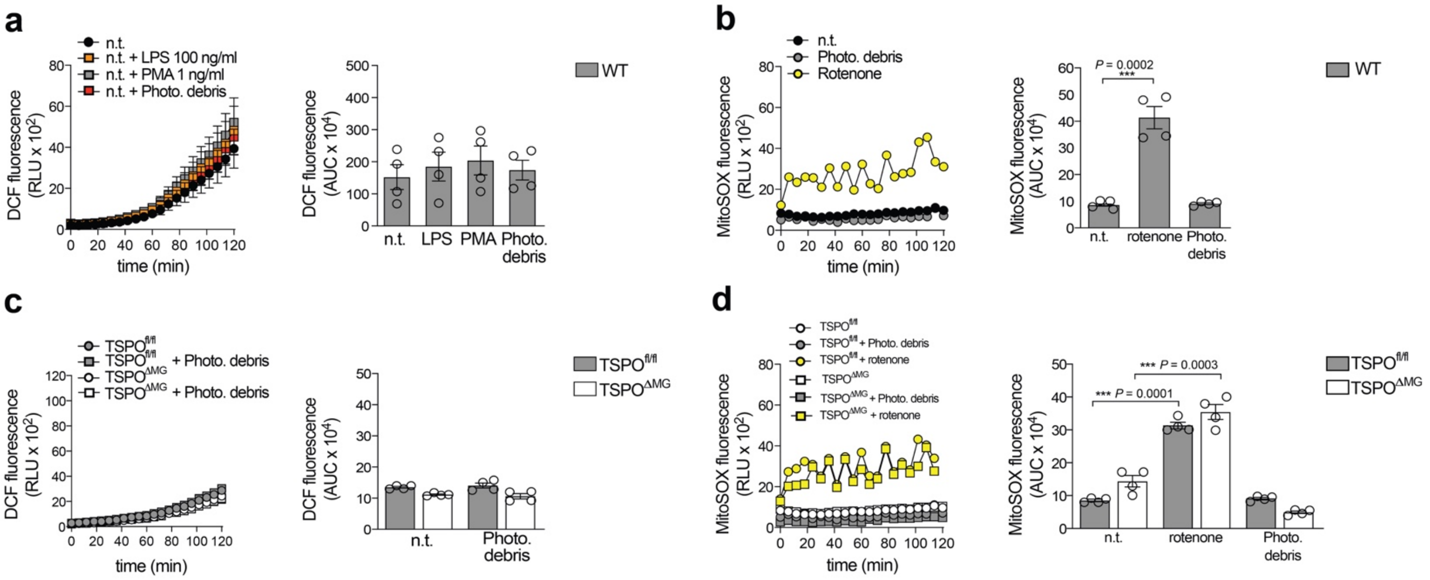

Supplementary Figure 4: Cytosolic ROS and matrix-derived ROS production are not induced in stimulated primary microglia. a-d Primary microglia were stimulated with LPS, PMA or photoreceptor cell debris. Kinetics of ROS production and the area under the curve (AUC) are shown. **a** Cytosolic ROS production; **b** matrix-derived ROS (mROS) production in WT mice. **c** Cytosolic ROS production; **d** mROS production in $TSPO^{fl/fl}$ and $TSPO^{\Delta MG}$ mice. Where indicated, ROS production into the mitochondrial matrix was induced with rotenone as a positive control. Data are presented as mean ± SEM. n= 4 independent experiments, two-tailed unpaired Student's *t* test, ****P*≤ 0.001. n.t., non-treated. Source data are provided as a Source Data file.

Supplementary Figure 5

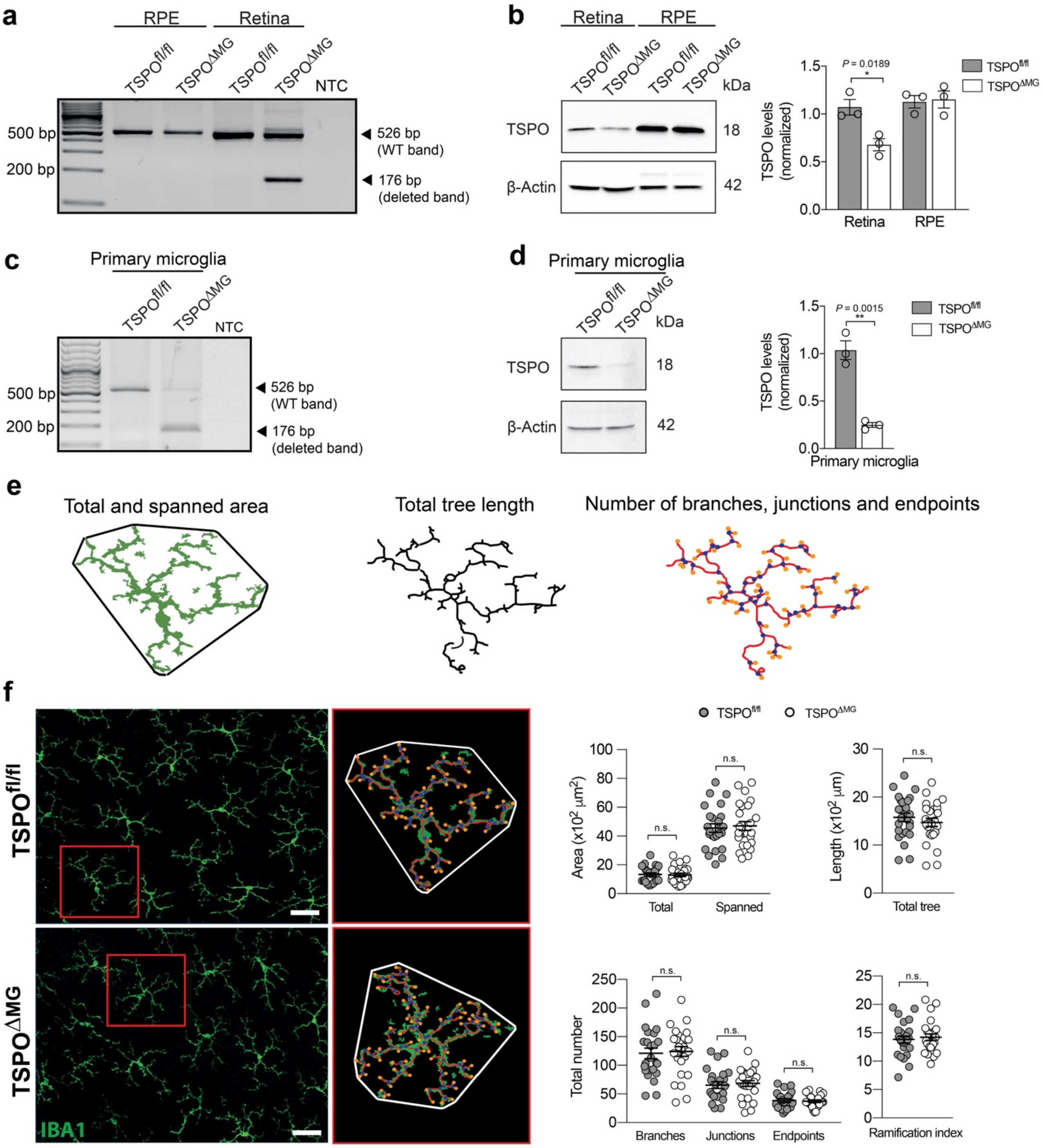

Supplementary Figure 5: Microglia of TSPO-KO mice exhibit a normal phenotype. a-d Validation of TSPO-KO in retina and RPE/choroid (**a, b**) and primary microglia (**c, d**). **a, c** Genomic PCR products spanning exon 1 and 4 of *Tspo*. WT band, 526 bp; *Tspo* deleted band, 176 bp; NTC, no template control. **b, d** TSPO protein levels and densitometric analysis of Western blots. TSPO signals were normalized to β-Actin. n= 3 retinas/RPEs from individual mice of three independent experiments. **e** Analysis of microglia phenotype in retinal flat mounts. Three morphological parameters were analyzed: 1) total (area of green

arbor) and spanned area (area circumscribed by the polygonal object defined by connecting the outer points of the dendritic arbor (green)); 2) total tree length (sum of all dendritic segments identified in the skeletonized arbor); 3) number of branches (blue dots), junctions (points where more than two branches meet, blue dots) and endpoints (orange dots) (identified in a skeletonized rendition of the arbor). **f** Skeleton analysis of microglia morphologies in Iba1-stained retinal flat mounts. Scale bar: 50 µm. Original photomicrographs (left panel) and skeletons (green) with associated convex hulls (white polygonal) (right panel). n= 25 microglia cells from individual retinas of three independent experiments. Data are presented as mean ± SEM; two-tailed unpaired Student's *t* test, *$P \leq 0.05$ and **$P \leq 0.01$. n.s., not significant. Source data are provided as a Source Data file.

Supplementary Figure 6

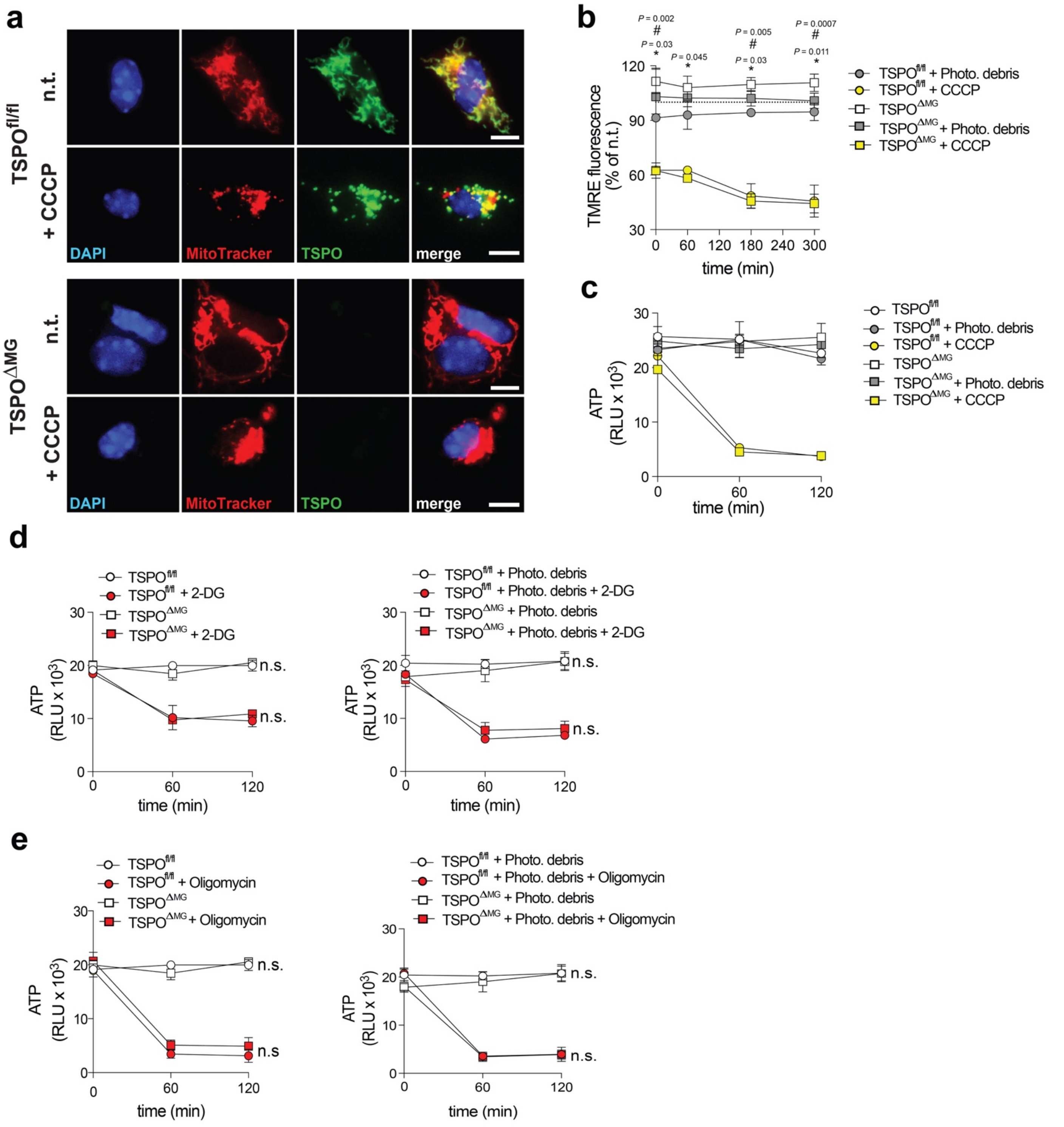

Supplementary Figure 6: TSPO-KO microglia exhibit a normal energy homeostasis. a Representative images of TSPO- and Mitotracker Red-stained mitochondria in $TSPO^{fl/fl}$ and $TSPO^{\Delta MG}$ primary microglia. Nuclei were counterstained with DAPI. Scale bar: 6 µm. **b** Analysis of mitochondrial membrane potential in $TSPO^{fl/fl}$ and $TSPO^{\Delta MG}$ primary microglia. Where indicated, $TSPO^{fl/fl}$ and $TSPO^{\Delta MG}$ primary microglia were stimulated with photoreceptor cell debris and mitochondrial membrane potential was impaired with CCCP as a positive control. n= 4 independent experiments; **c-e** Analysis of total ATP levels.

Where indicated, primary microglia from $TSPO^{fl/fl}$ and $TSPO^{\Delta MG}$ mice were stimulated with photoreceptor cell debris and treated either with 200 µM CCCP **(c)** to impair the mitochondrial membrane potential, 500 µM 2-Deoxy-D-glucose (2-DG) to inhibit glycolysis **(d)** or 10 µM oligomycin to inhibit complex V of the ETC **(e)**. n= 3 independent experiments. Data are presented as mean ± SEM; two-tailed unpaired Student's *t* test; *P< 0.01 when $TSPO^{fl/fl}$ compared to $TSPO^{fl/fl}$ + Photo. debris; $^{\#}P$< 0.01 when $TSPO^{fl/fl}$ compared to $TSPO^{\Delta MG}$. n.s., not significant. n.t., non-treated. Source data are provided as a Source Data file.

Supplementary Figure 7

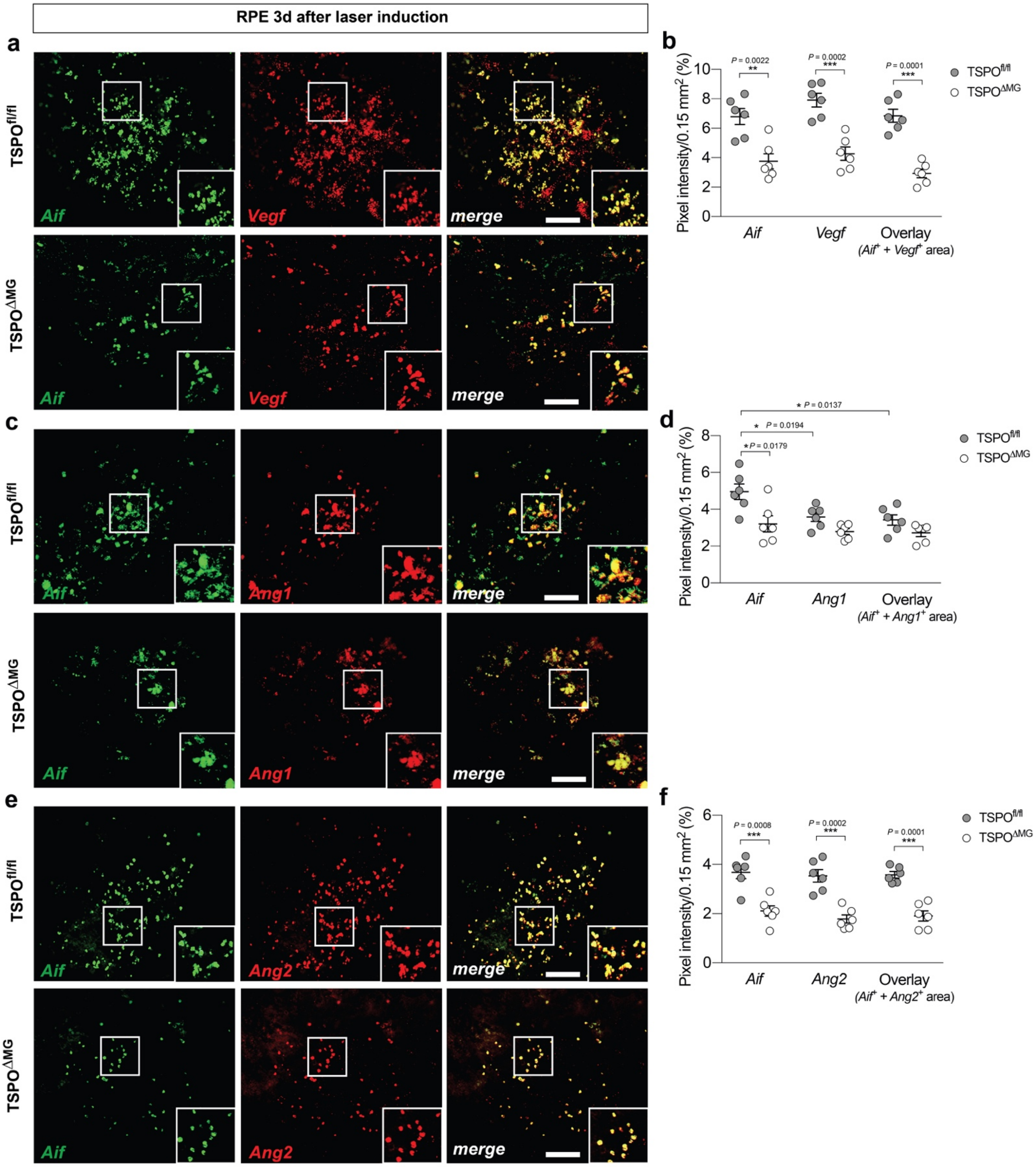

Supplementary Figure 7: Reactive mononuclear phagocytes express *Vegf, Ang1* and *Ang2* mRNAs in laser lesions. Representative images of *in situ* hybridization of individual laser lesions in RPE/choroidal flat mounts of TSPO$^{fl/fl}$ and TSPO$^{\Delta MG}$ mice at 3 days post laser using mRNA probes for *Aif* to label MNPs in combination with *Vegf* **(a)**, *Ang1* **(c)**, *Ang2* **(e)**. Scale bar: 50 μm. Inlays show higher magnification.

Analysis of *in situ* hybridization signals of *Aif* together with *Vegf* **(b)**, *Ang1* **(d)** and *Ang2* **(f)** per laser spot in RPE/choroidal flat mounts 3 days after laser coagulation by quantifying the percentage of colored pixels per area. Data are presented as mean ± SEM. n= 6 laser spots; two-tailed unpaired Student's *t* test, **P*< 0.05, ***P*< 0.01 and ****P*≤ 0.001. *Aif*, Allograft inflammatory factor 1. Source data are provided as a Source Data file.

Supplementary Figure 8

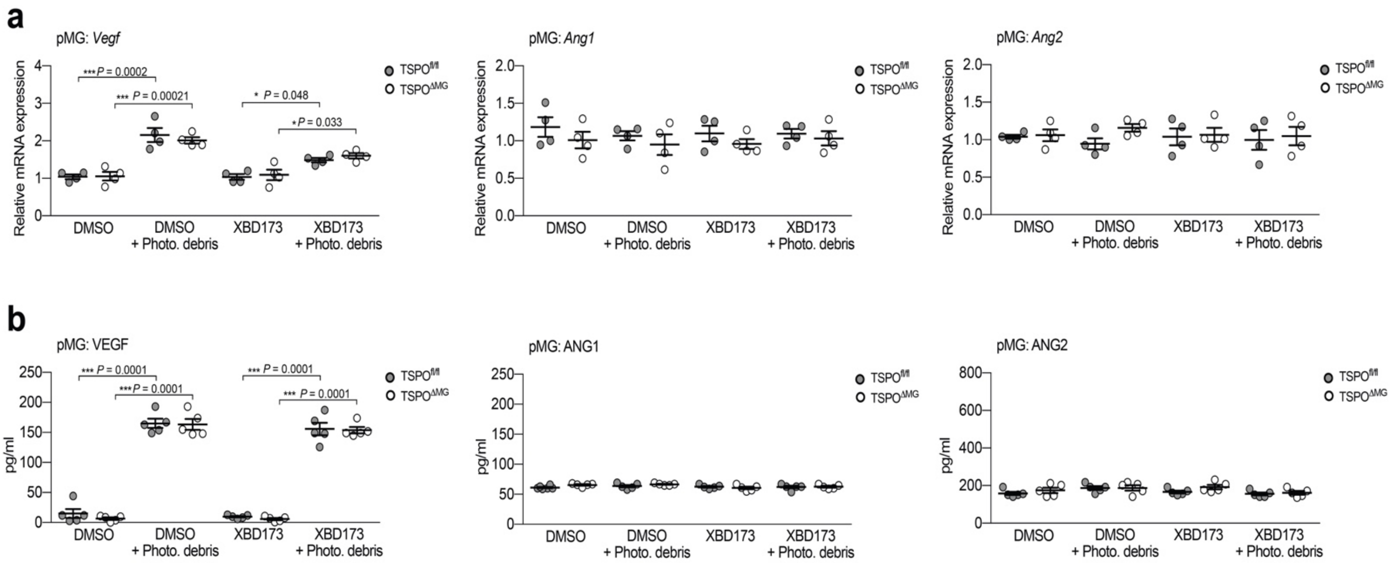

Supplementary Figure 8: Stimulated primary microglia show increased levels of VEGF but not ANG1 and ANG2. a *Vegf*, *Ang1* and *Ang2* mRNA levels in pMG from $TSPO^{fl/fl}$ and $TSPO^{\Delta MG}$ mice 6h after stimulation with photoreceptor cell debris. n= 4 independent experiments. Linear mixed model was used for statistical analyses; **P*< 0.05 and ****P*≤ 0.001. **b** Pro-angiogenic growth factor levels in pMG from $TSPO^{fl/fl}$ and $TSPO^{\Delta MG}$ mice 6h after stimulation with photoreceptor cell debris. n= 5 independent experiments. Data are presented as mean ± SEM. Linear mixed model was used for statistical analyses; ****P*≤ 0.001. Source data are provided as a Source Data file.

Supplementary Figure 9

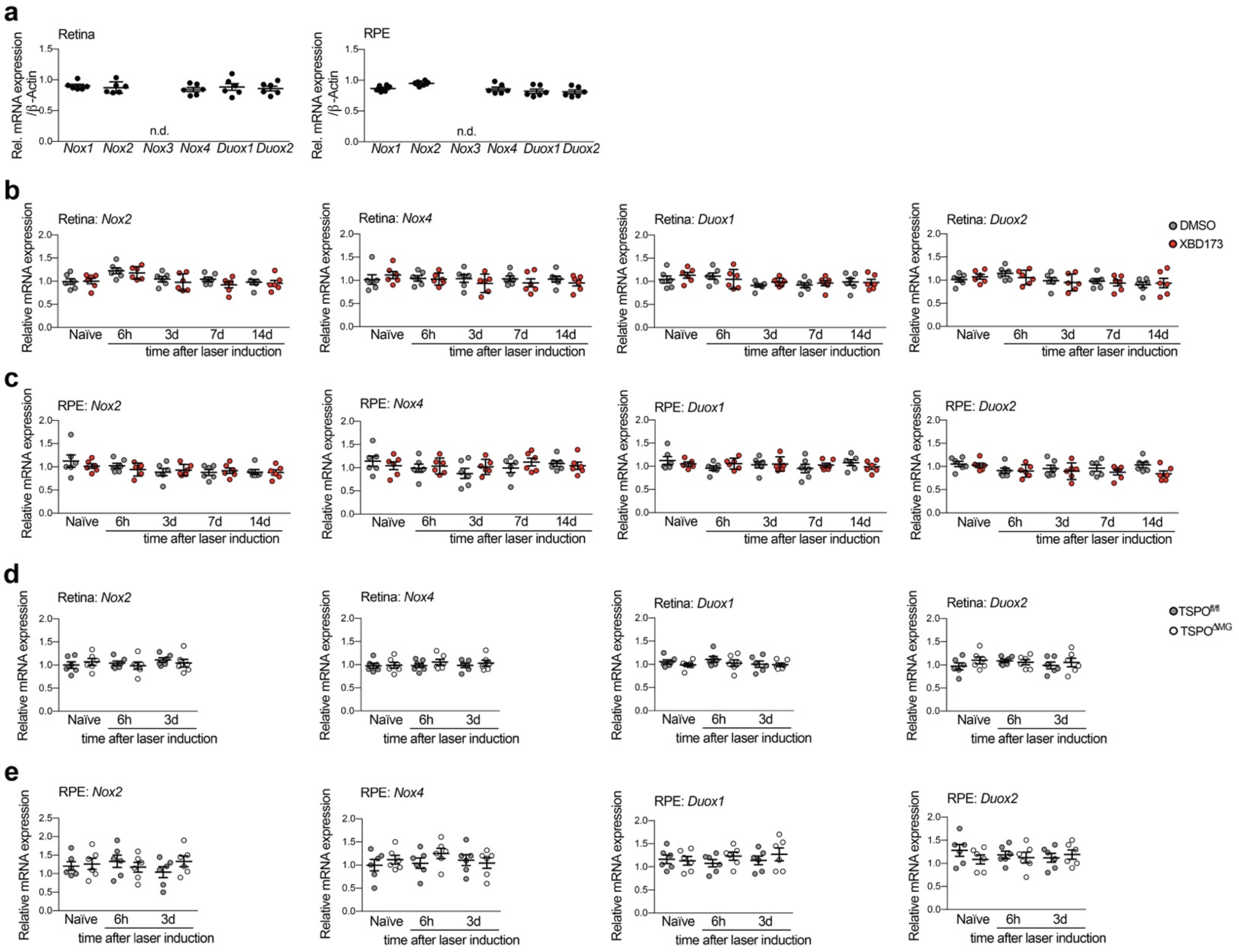

Supplementary Figure 9: NADPH oxidase (NOX) family enzymes *Nox2*, *Nox4*, *Duox1* and *Duox2* do not show a laser-induced gene expression in mice. a Relative expression of NOX family members in WT retina and RPE/choroid. Transcript levels for each enzyme were normalized to β-Actin. **b, c** Relative expression of NOX family members in the retina (**b**) and RPE/choroid (**c**) after laser-induced CNV in DMSO- or XBD173-treated mice. **d, e** Relative expression of NOX family members in the retina (**d**) and RPE/choroid (**e**) after laser-induced CNV in $TSPO^{fl/fl}$ and $TSPO^{\Delta MG}$ mice. Data are presented as mean ± SEM. n= 6 retinas/RPEs from individual mice. Linear mixed model was used for statistical analyses. N.d., not detected. Source data are provided as a Source Data file.

Supplementary Figure 10

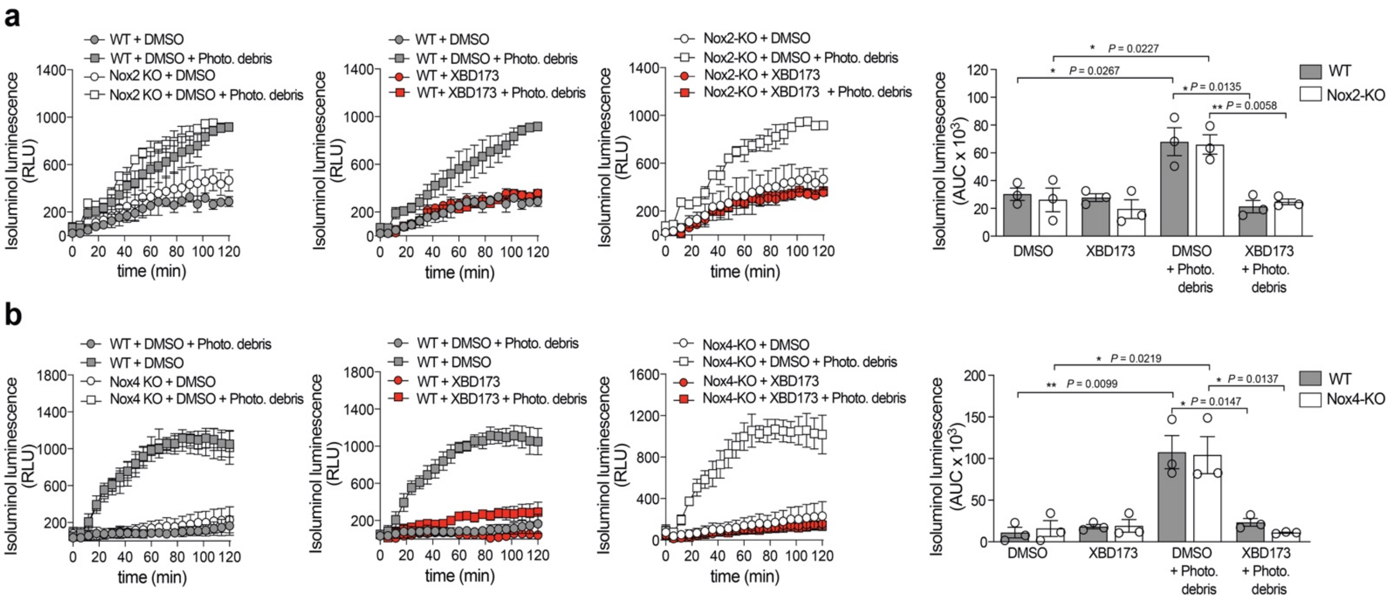

Supplementary Figure 10: Photoreceptor cell debris does not induce ROS production by NOX2 and NOX4. a Quantification of extracellular ROS production by primary microglia from WT and Nox2-KO mice. **b** Quantification of extracellular ROS production by primary microglia from WT and Nox4-KO mice. Kinetics of ROS production and the area under the curve (AUC) are shown. Data are presented as mean ± SEM. n= 3 independent experiments, two-tailed unpaired Student's *t* test, $*P < 0.05$ and $**P < 0.01$. Source data are provided as a Source Data file.

Supplementary Figure 11

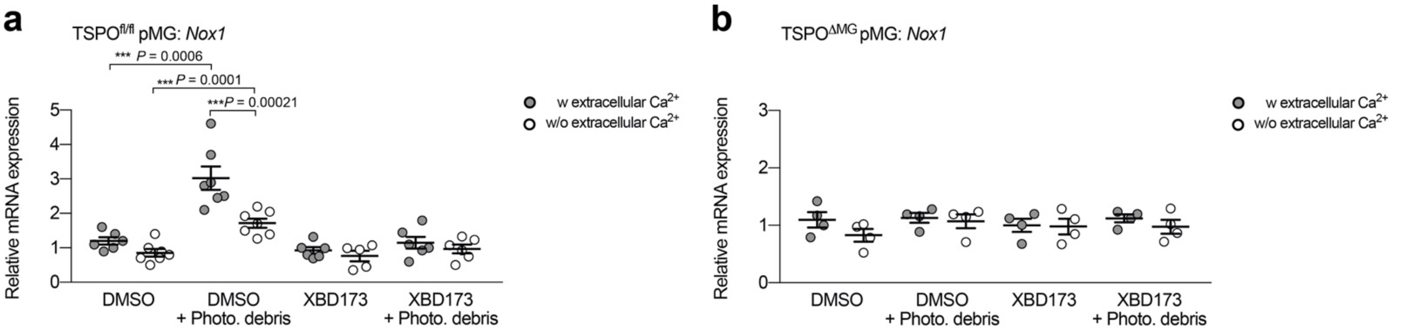

Supplementary Figure 11: Lack of extracellular Ca^{2+} reduces expression of *Nox1* in primary microglia. *Nox1* mRNA levels in levels in pMG from $TSPO^{fl/fl}$ **(a)** and $TSPO^{\Delta MG}$ **(b)** mice 6h after stimulation with photoreceptor cell debris in the presence or absence of extracellular Ca^{2+}. Data are presented as mean ± SEM. n= 6 for $TSPO^{fl/fl}$ pMG and n= 4 independent experiments for $TSPO^{\Delta MG}$ pMG. Linear mixed model was used for statistical analyses, ***$P \leq 0.001$. W/o; without; w, with. Source data are provided as a Source Data file.

Supplementary Figure 12

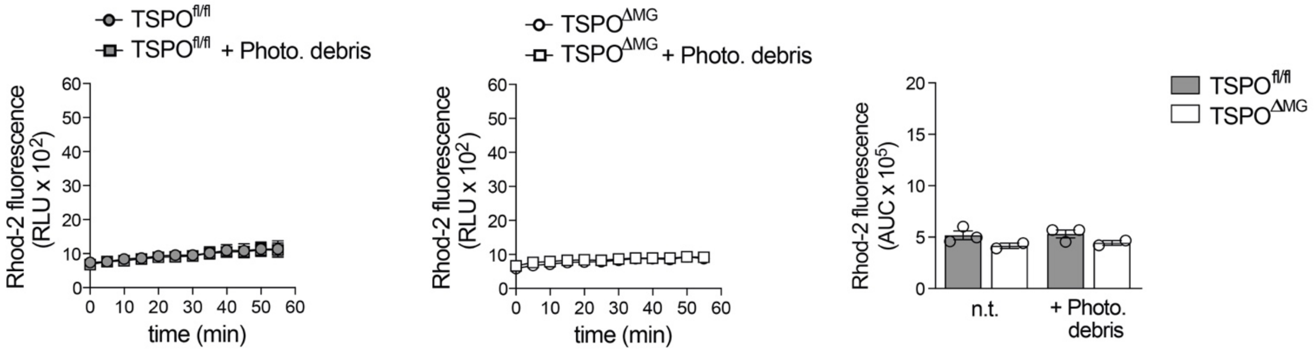

Supplementary Figure 12: Photoreceptor cell debris does not increase mitochondrial Ca^{2+} levels. Quantification of mitochondrial calcium levels in primary microglia from $TSPO^{fl/fl}$ and $TSPO^{\Delta MG}$ mice**.** Where indicated, primary microglia were stimulated with photoreceptor cell debris. Kinetics of calcium measurements and the area under the curve (AUC) are shown. Data are presented as mean ± SEM. n= 3 independent experiments, two-tailed unpaired Student's *t* test. Source data are provided as a Source Data file.

Supplementary Figure 13

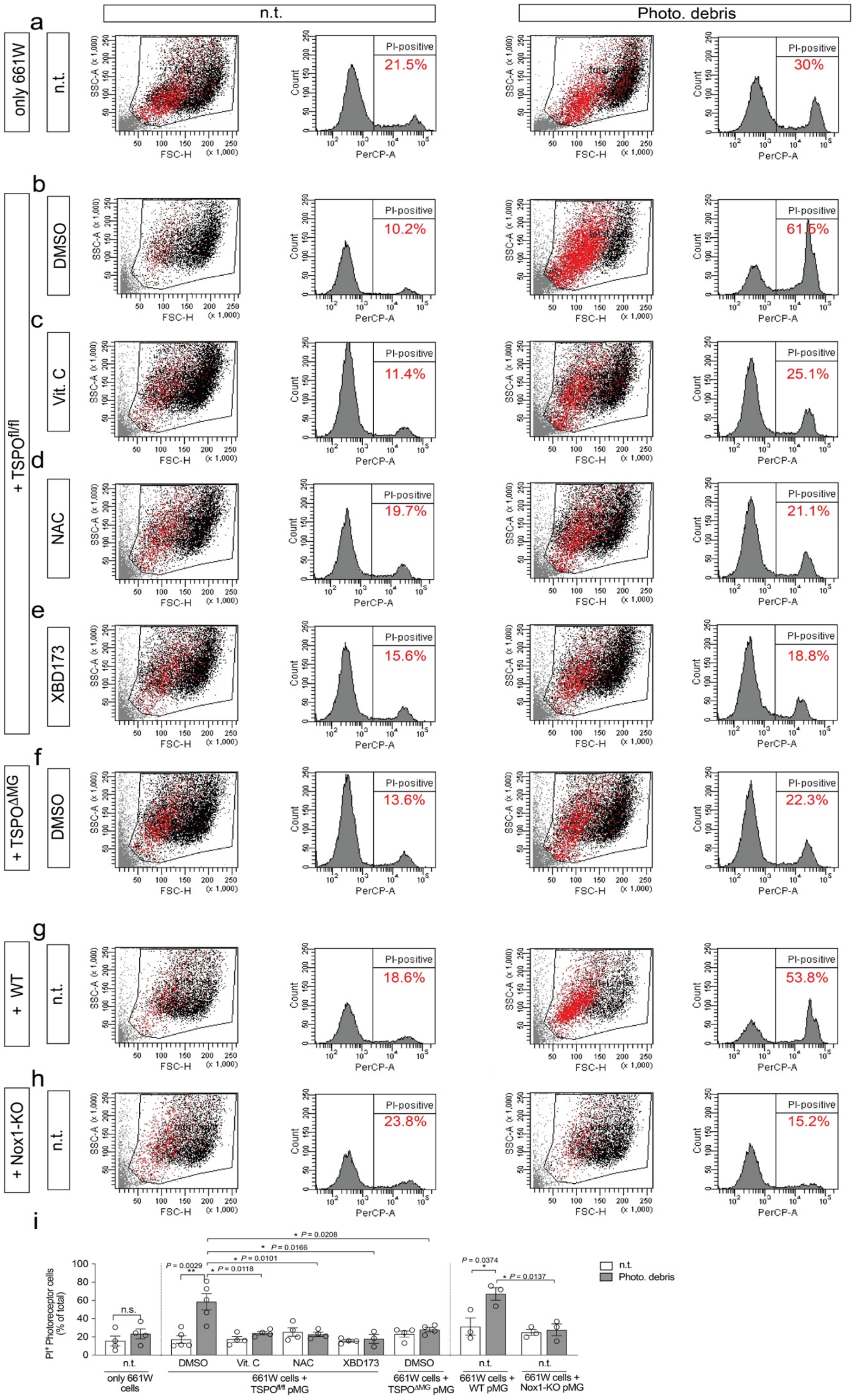

Supplementary Figure 13: Extracellular ROS damage photoreceptor cells in a paracrine manner. ROS-induced cell death of 661W photoreceptor cells alone **(a)** or in co-culture with microglia **(b-h)** was determined by analyzing the percentage of PI^{+}-photoreceptor cells isolated from the trans-well inlays. Photoreceptor cells and microglia were unstimulated (right panel) or treated with photoreceptor cell debris (right panel) to induce ROS production in microglia. Photoreceptor cells were co-cultured with $TSPO^{fl/fl}$ microglia treated with the vehicle control **(b)** or with $TSPO^{fl/fl}$ microglia in medium with Vitamin C (Vit. c) **(c)**, NAC **(d)** or XBD173 **(e)** or with $TSPO^{\Delta MG}$ microglia **(f)**. Photoreceptor cells were co-cultured with corresponding WT microglia **(g)** or Nox1-KO microglia **(h)**. Bar chart showing the quantification of photoreceptor cell death **(i)**. 5,000 cells were counted per sample. Gating strategy: detached 661W photoreceptor cells were gated based on FSC-H/SSC-A to exclude cell debris. Cell death in the gated population was analyzed by detection of PI-positive 661W photoreceptor cells based on PerCP-A fluorescence. In each experiment 661W photoreceptor cells cultured alone served as negative control groups. Data are shown as mean ± SEM, n= 4 independent experiments ($TSPO^{fl/fl}$ DMSO n= 5; XBD173 + Photo. debris n= 3; Nox1 WT and KO n= 3 independent experiments), two-tailed unpaired Student's *t* test, *$P \leq 0.05$ and **$P \leq 0.01$.n.s, not significant; n.t. = non-treated. Source data are provided as a Source Data file.
